# Oncotype DX results increase concordance in adjuvant chemotherapy recommendations for early-stage breast cancer

**DOI:** 10.1038/s41523-023-00559-6

**Published:** 2023-06-08

**Authors:** Luca Licata, Giulia Viale, Mario Giuliano, Giuseppe Curigliano, Mariana Chavez-MacGregor, Julia Foldi, Oluchi Oke, Joseph Collins, Lucia Del Mastro, Fabio Puglisi, Filippo Montemurro, Claudio Vernieri, Lorenzo Gerratana, Sara Giordano, Alessia Rognone, Lorenzo Sica, Oreste Davide Gentilini, Stefano Cascinu, Lajos Pusztai, Antonio Giordano, Carmen Criscitiello, Giampaolo Bianchini

**Affiliations:** 1 Department of Medical Oncology, San Raffaele Hospital, Milan, Italy; 2 School of Medicine and Surgery, Vita-Salute San Raffaele University, Milan, Italy; 3 Department of Clinical Medicine and Surgery, University of Naples Federico II, Naples, Italy; 4 Division of New Drugs and Early Drug Development, European Institute of Oncology, IRCCS, Milan, Italy; 5 Department of Oncology and Hemato-Oncology, University of Milan, Milan, Italy; 6 Departments of Breast Medical Oncology and Health Services Research, The University of Texas MD Anderson Cancer Center, Houston, TX USA; 7 Section of Medical Oncology, Yale School of Medicine, New Haven, CT USA; 8 Department of General Oncology, The University of Texas MD Anderson Cancer Center, Houston, TX USA; 9 Oncology Associates, South Carolina, Columbia, SC USA; 10 Department of Internal Medicine and Medical Specialties (DiMI), School of Medicine, University of Genova, Genova, Italy; 11 Department of Medical Oncology, Clinical Unit of Medical Oncology, IRCCS Hospital Policlinico San Martino, Genova, Italy; 12 Department of Medical Oncology, Unit of Medical Oncology and Cancer Prevention, Centro di Riferimento Oncologico di Aviano (CRO) IRCCS, Aviano, Italy; 13 Department of Medicine (DAME), University of Udine, Udine, Italy; 14 Breast Surgery Strategic Program, Candiolo Cancer Institute, Fondazione del Piemonte per l’Oncologia - IRCCS, Torino, Italy; 15 Breast Unit, Fondazione IRCCS Istituto Nazionale dei Tumori, Milano, Italy; 16 IFOM ETS - the AIRC Institute of Molecular Oncology, Milan, Italy; 17 Department of Medical Oncology, Aviano Oncology Reference Center (IRCCS), Aviano, Italy; 18 Dana-Farber Cancer Institute, Harvard Medical School, Boston, MA USA; 19 Breast Surgery Unit, San Raffaele Hospital, Milan, Italy; 20 Yale Cancer Center, Yale School of Medicine, New Haven, CT USA

**Keywords:** Breast cancer, Breast cancer

## Abstract

Adjuvant chemotherapy recommendations for ER+/HER2− early-stage breast cancers (eBC) involve integrating prognostic and predictive information which rely on physician judgment; this can lead to discordant recommendations. In this study we aim to evaluate whether Oncotype DX improves confidence and agreement among oncologists in adjuvant chemotherapy recommendations. We randomly select 30 patients with ER+/HER2− eBC and recurrence score (RS) available from an institutional database. We ask 16 breast oncologists with varying years of clinical practice in Italy and the US to provide recommendation for the addition of chemotherapy to endocrine therapy and their degree of confidence in the recommendation twice; first, based on clinicopathologic features only (pre-RS), and then with RS result (post-RS). Pre-RS, the average rate of chemotherapy recommendation is 50.8% and is higher among junior (62% vs 44%; *p* < 0.001), but similar by country. Oncologists are uncertain in 39% of cases and recommendations are discordant in 27% of cases (interobserver agreement K 0.47). Post-RS, 30% of physicians change recommendation, uncertainty in recommendation decreases to 5.6%, and discordance decreases to 7% (interobserver agreement K 0.85). Interpretation of clinicopathologic features alone to recommend adjuvant chemotherapy results in 1 out of 4 discordant recommendations and relatively high physician uncertainty. Oncotype DX results decrease discordancy to 1 out of 15, and reduce physician uncertainty. Genomic assay results reduce subjectivity in adjuvant chemotherapy recommendations for ER +/HER2− eBC.

## Introduction

Adjuvant therapy decision-making for patients with early breast cancer is based on several considerations, including estimation of risk of recurrence, expected benefit from various components of treatment, patients’ preferences, and probabilities of short- and long-term toxicities.

Among women with estrogen receptor-positive/human epidermal growth factor receptor 2-negative (ER+/HER2−) early breast cancer, adjuvant endocrine therapy is highly effective^1^. The addition of chemotherapy has also demonstrated a reduction in the risk of recurrence and death in selected patients^2,3^ and, more recently, the CDK4/6 inhibitor abemaciclib has been shown to further reduce the risk of recurrence in high-risk patients^4^. In modern oncology one size does not fit all and optimal treatment tailoring to minimize both overtreatment and undertreatment without compromising survival rate has been (and still is) a major focus of research.

Advances in our understanding of the molecular biology of breast cancer have led to the development of genomic assays that help identify patients who can be safely spared adjuvant chemotherapy^5^. Among genomic assays, the 21-gene recurrence score (RS) assay (Oncotype DX) is one of the most widely used. Oncotype DX provides prognostic information independent of clinicopathologic features and predicts chemotherapy benefit in patients with node-negative and node-positive (1–3 nodes) ER+/HER2− early breast cancer^6–8^. The clinical utility of Oncotype DX has been prospectively evaluated in the large West German Study Group PlanB, TAILORx, and RxPONDER randomized clinical trials^9–11^, achieving a level of evidence and a category of recommendation of IA^3,12^. More recently, an effort to provide an estimation of the absolute chemotherapy benefit expected in individual patients led to the development of the RSClin tool that combines prognostic information from tumor size, grade and age with RS to provide a more accurate prognostic risk estimate^13^. Multiple studies showed that the use of the Oncotype DX assay results leads to changes in treatment recommendations by physicians leading to decrease in adjuvant chemotherapy prescription^14–16^. The use of the assay is cost-effective under most circumstances^17^, and it increases physician and patient confidence in treatment recommendations^18,19^. It is usually implied that genomic assays also reduce unwarranted subjectivity in treatment recommendations. However, to what extent RS results impact concordance of physician recommendations has not been studied in the past.

The goal of this study is to use real-life case histories to assess how physician confidence in adjuvant chemotherapy recommendation and concordance in the recommendations change by the RS results, and if physicians’ years of experience or country of practice have an effect on RS interpretation.

## Results

Among 30 patients included in the analysis, median age was 50.5 years (range 30–75) and 40% were premenopausal. Most patients (83%) had invasive ductal carcinoma; half had a primary tumor >20 mm in size (pT2); 27% had grade 3 tumors, 90% a Ki67 level >20%, and 40% a PgR expression ≤20% (Table 1).

**Table 1:** Patient and disease baseline characteristics.

Characteristic	Overall	Recurrence score categories	
		0–25 (%)	26–100 (%)
Age			
Median - yr	50.5		
Range -yr	30–75		
Menopausal status			
Pre-/perimenopausal	12 (40)	7 (58)	5 (42)
Postmenopausal	18 (60)	13 (72)	5 (28)
Histology			
Ductal	25 (83)	16 (64)	9 (36)
Lobular	4 (13)	3 (75)	1 (25)
Mucinous	1 (4)	1 (100)	0
Primary tumor			
T1c	15 (50)	9 (60)	6 (40)
T2	15 (50)	11 (73)	4 (27)
Grade			
G2	22 (73)	17 (77)	5 (23)
G3	8 (27)	3 (37)	5 (63)
Ki67			
≤20	3 (10)	2 (67)	1 (33)
21–30	19 (63)	15 (79)	4 (21)
>30	8 (27)	3 (37)	5 (63)
PgR			
≤20	12 (40)	6 (50)	6 (50)
>20	18 (60)	14 (78)	4 (22)

Based on the clinicopathologic characteristics only, the average rate of chemotherapy recommendation was 51% (range 26.7–76.7%). Chemotherapy prescription rate was higher among Junior than Senior physicians (62% vs 44%; *p* < 0.001; chi-square test), but similar by country (US 50%, Italy 51%). Comparing pre- and post-RS chemotherapy recommendations by the same physician, overall 30% of recommendations (142 of 480) were changed by the assay result, with no significant differences by experience or country (28% vs 33% for Junior and Senior, respectively; 27% vs 33% for Italy and US, respectively) (Supplementary Fig. 1). Across the group of physicians, treatment recommendations for individual patients changed from 6 to 15 of the 30 patients that were assessed. In 20% of cases (94 of 480), the change in treatment led to chemotherapy omission, with a range of 1–11 out of 30 recommendations across oncologists. In 10% (48 of 480), the change led to the addition of chemotherapy to adjuvant endocrine therapy, with a range of 0–7 out of 30 recommendations given by each oncologist.

### Confidence

**Fig. 1:**
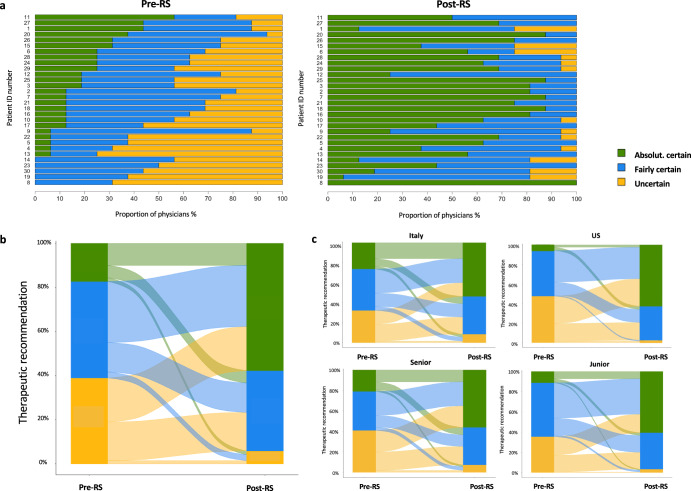
Change of confidence in the therapeutic decision after RS. **a** Confidence in the therapeutic decision pre- and post- RS results for each case. **b** Change of confidence in therapeutic recommendations pre- and post- RS results in the overall population. **c** Change of confidence in therapeutic recommendations pre- and post- RS results by oncologist country and experience. In (**b** and **c**), *Y* axes indicate the degree of confidence for all the therapeutic recommendations provided by each oncologist for each patient. Alluvial plots describe the change of confidence for each individual therapeutic recommendation pre- and post- RS in the overall population (**b**) or by oncologist country and experience (**c**).

Oncologists providing their recommendations pre-RS results were uncertain, fairly certain, and absolutely certain in 39%, 44%, and 17% of the cases, respectively. Uncertainty was significantly higher among US oncologists than among Italian oncologists (48% vs 32%, p 0.0008; chi-square test), while there were no significant differences between Junior and Senior (36% vs 41%, p 0.28) (Fig. 1).

Oncologists providing their recommendations post-RS were uncertain, fairly certain, and absolutely certain in 6%, 36%, and 58% of the cases, respectively. Uncertainty was significantly higher among Italian oncologists than among US oncologists (9% vs 2%, *p* 0.008; chi-square test), while numerical differences between Senior and Junior were not significant (7% vs 3%, *p* 0.11; chi-square test) (Fig. 1). Overall, 82% and 79% of the post-RS recommendations given with uncertainty were provided by Italian and Senior oncologists, respectively.

Post-RS, confidence increased and decreased in 65% and 10%, respectively (*p* < 0.001; chi-square test). The confidence increase was significantly higher among US oncologists compared to Italian oncologists (79% US vs 55% Italy, *p* < 0.00001; chi-square test), while no significant differences in the confidence increase were observed between Senior and Junior (Supplementary Fig. 2). Confidence decrease was almost confined to Italian oncologists (14% Italian vs 4% US). Interestingly, in 11.5% of the cases the recommendation pre-RS was given with absolute/fair certainty, and— after getting RS result—the recommendation was changed again with absolute/fair certainty.

### Agreement

**Fig. 2:**
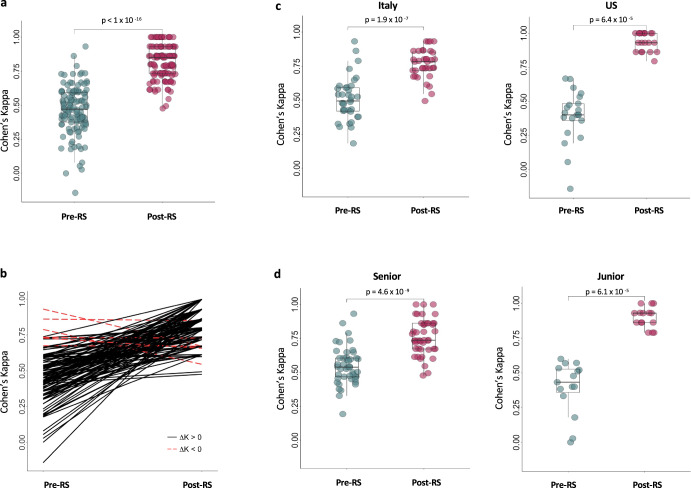
Overall agreement in the therapeutic indication given pre- and post-RS. **a** Cohen’s kappa coefficients for pairwise inter-observer agreement on therapeutic recommendation pre- and post-RS result. **b** Change in Cohen’s kappa pre- and post-RS results for each pairwise comparison. **c**, **d** Cohen’s kappa coefficients for pairwise inter-observer agreement pre- and post-RS result by oncologist country (**c**) and experience (**d**). In (**a**, **c** and **d**), each dot represents the result of a pairwise comparison between two oncologists for every patient. The *p* values (Wilcoxon test) refer to the comparison between the overall agreement pre-RS and post-RS. The horizontal lines in the boxes denote the first quartile, median, and third quartile. The boundaries of the whiskers are based on the 1.5 × interquartile values.

The interobserver agreement on chemotherapy recommendation pre-RS was only moderate (K 0.47, range −0.14–0.93; FK 0.46), corresponding to 27% (53.3–3.3%) discordant recommendations. Pre-RS agreement was significantly lower among Junior compared to Senior (Junior K 0.39 vs Senior K 0.54; Wilcoxon *p* value: 0.008) and among US oncologists compared to Italian (US K 0.39 vs Italian K 0.51; Wilcoxon *p* value: 0.04) (Fig. 2). Post-RS, the interobserver agreement was near perfect (K 0.85, range 0.47–1.00), corresponding to only 7% (26.7–0%) discordant recommendations. Post-RS agreement was significantly higher in US (US K 0.94 vs Italian K 0.77; Wilcoxon *p* value < 0.001) and Junior oncologists (Junior K 0.90 vs Senior K 0.76; Wilcoxon *p* value < 0.001) (Fig. 2). The intraobserver agreement pre- and post-RS was only fair (K 0.40; range 0.05–0.60). Results of agreement comparison using Fleiss’ kappa were similar.

### An illustrative case

A few cases accounted for the highest discrepancy and uncertainty. Among these, one offers the opportunity to investigate potential clinicopathologic characteristics associated with uncertainty and discordancy. Patient number 19 was a 41-year-old premenopausal woman diagnosed with a node-negative invasive ductal carcinoma of 2.1 cm. Biological characteristics were: Grade 2, ER 90%, PgR 80%, Ki67 26%. Before knowing the RS result, oncologists split into two groups: half (4 Italy, 3 Junior and 1 Senior; 4 US, 2 Junior and 2 Senior) recommended the addition of chemotherapy to adjuvant endocrine therapy, the other half recommended endocrine therapy alone. In providing these recommendations, 63% of the oncologists were uncertain (5 Italy, 1 Junior and 4 Senior; 5 US, 2 Junior and 3 Senior) and 37% were fairly certain. No one was absolutely certain. After knowing the RS result of 24, most of the oncologists (88%) recommended the addition of chemotherapy to adjuvant endocrine therapy and uncertainty decreased to 19% (3 Senior, 2 Italy and 1 US). Among the 8 oncologists who changed their recommendation, the confidence increased in 4 cases and remained stable in 4.

## Discussion

Decisions around adding chemotherapy to adjuvant endocrine therapy in patients with ER+/HER2− early breast cancer can be challenging.

While recommending endocrine therapy is straightforward for ER positive cancers, there are no clinicopathologic features clearly and independently predictive of chemotherapy sensitivity. Hence, physician recommendations to add adjuvant chemotherapy to endocrine therapy, or not, is based on subjective estimates of risk of distant recurrence and presumptions about chemotherapy responsiveness.

Due to the subjective weighting of clinicopathologic features that collectively determine risk of recurrence, different oncologists often provide different adjuvant chemotherapy recommendations for the same cases, and are also well aware of the uncertainty in their decisions. Moreover, some of the clinicopathologic features are subject to technical reproducibility issues, intralaboratory and interlaboratory, increasing the uncertainty for chemotherapy recommendations. Guidelines on the management of early breast cancer provide general recommendations and guidance, but allow room for physician judgement and patient preference. This is especially true for cases with controversial clinicopathologic characteristics that may fall in a “gray zone” for chemotherapy recommendations.

Genomic signatures such as Oncotype DX, despite being affected by reproducibility issues related to intratumor heterogeneity, provide complementary information to clinicopathologic prognostic variables and may aid clinicians in more accurately identifying patients with good outcomes for whom chemotherapy can be safely omitted^13^. Indeed, a plethora of studies demonstrated that the use of genomic signatures can lead to a decrease of chemotherapy recommendation in up to 50% of cases^19–28^, and some studies showed that physicians’ confidence in treatment recommendations may improve with the use of these signatures^19–21,23^.

Discordant adjuvant chemotherapy recommendations for the same case by different physicians is commonly encountered in routine practice and is well documented in the literature. This is distressing for patients, generally undermines trust in the health care system and results in unwarranted variance in practice. Undoubtedly, it also results in under- and overtreatment of some patients. It is assumed by practice guidelines that the use of genomic assays would reduce heterogeneity in practice and variation in treatment recommendations. However, how genomic test results affect concordance in physician treatment recommendations has not been studied. We show that Oncotype DX assay results significantly decreased discordant adjuvant chemotherapy recommendations for real cases even among a group of academic breast cancer experts. In the absence of the RS result, on average 1 out of 4 patients received discordant recommendations, while discordant recommendations were observed in only 1 out of 15 patients with RS available.

Of note, all participating oncologists in the study belonged to tertiary institutions for breast cancer treatment, and with RS results the interobserver agreement significantly increased irrespective of oncologists’ country and experience.

Our study also showed that the use of Oncotype DX significantly reduced the degree of physicians’ uncertainty about the role of adjuvant chemotherapy. Pre-RS, the degree of confidence was lower among US oncologists compared to Italian ones, but post-RS we observed exactly the opposite: uncertainty was lower among US oncologists and was almost confined to Italian ones. This indicates that US physicians had greater “trust” in the RS result than their Italian counterparts. This may be due to greater availability of the assay and larger number of studies conducted with Oncotype DX in the USA. Oncotype DX has been commercially available in US since 2004, and its use among US oncologists has progressively increased^29^. In Italy, Oncotype DX and other genomic tests are only being reimbursed by the National Health Service since May 2021^30^, and their use at a national level is currently quite scattered.

Notably, we showed that the use of Oncotype DX significantly increased confidence and agreement among Junior oncologists. In this group, the agreement pre-RS was only fair (FK 0.39) and significantly lower compared to Senior. Post-RS, the agreement was near perfect (FK 0.93). These data suggest that in less experienced oncologists the role of Oncotype DX in aiding decision making may be even more important.

The illustrative clinical case we presented offers the opportunity to investigate the clinicopathologic characteristics associated with higher uncertainty and discordancy. Young age, premenopausal status and controversial biological characteristics (high hormone receptors expression and intermediate/high proliferation) had split the participating oncologists exactly in half when asked for the addition or not of adjuvant chemotherapy, and none of them provided the recommendation with absolute certainty. After knowing the RS result, 88% of the oncologists converged to the addition of adjuvant chemotherapy and only 3 remained uncertain in providing their recommendation. This example points out the uncertainties around the optimal adjuvant therapy in young women with low genomic risk, where some of the benefit observed with chemotherapy might be due to its endocrine effect of ovarian function suppression, and highlights the importance of the careful interpretation of genomic test results within the scope of a comprehensive evaluation that includes also the clinicopathologic variables.

The most peculiar and novel element of our study is that Oncotype Dx, besides its recognized and established role in fine-tuning treatment recommendations, reduces the differences in treatment choice among oncologists, who often give discordant recommendations in the absence of a genomic test. This aspect is highly valuable, as it guarantees homogeneous treatments to patients across different institutions. Such a result is even more valuable in an era in which patients may ask for a second opinion. One could argue that tools other than oncotype, such as online available risk calculators, might be also useful in reducing discordancy in adjuvant therapy recommendations if widely used by clinicians. However, since none of these tools are recommended by Guidelines to tailor adjuvant therapy decisions, they are not widely employed in clinical practice and no studies have investigated their role in this context.

In conclusion, we showed that Oncotype DX significantly increased physicians’ confidence in adjuvant chemotherapy recommendations and agreement among oncologists in providing adiuvant treatment recommendations for patients with ER+/HER2− early breast cancer. In our opinion, these are two highly relevant and underappreciated benefits of using genomic tests in routine practice. These results add to a large body of literature that supports the use of genomic assays to determine adjuvant chemotherapy use and encourage a broader implementation of these assays in clinical practice.

## Materials and methods

### Clinical cases

The study used real-world data retrieved from the institutional database of San Raffaele Hospital in Milan, Italy. Among patients with ER+/HER2− early breast cancer who underwent breast surgery at our institution, we identified those with stage pT1c-2, node-negative, grade 2/3, Ki67 of at least 15%, and RS available. Among patients with the above characteristics, we randomly selected 30 cases.

### Procedures

Within our network of collaborating colleagues, we identified 16 breast oncologists with different years of clinical practice experience in Italy and US: 10 seniors, defined as oncologists with at least 15 years of experience (Italy, *n* = 6; US, *n* = 4), and 6 juniors, defined as oncologists within 2 years from the end of fellowship (Italy, *n* = 3; US, *n* = 3). Each participant was contacted by email and received a file containing the clinicopathologic features of the 30 patients: age, menopausal status, tumor histotype, tumor size, grade, ER, PgR and Ki67 levels (Supplementary Table 1). The study was conducted between January and March 2020.

Participants had to provide recommendations if adjuvant chemotherapy was indicated or not, twice; first, based on clinicopathologic features only (pre-RS), and then (about 2 weeks later) with the RS results available (post-RS). Each pre- and post-RS adjuvant chemotherapy recommendation had to be annotated with three levels of confidence; absolutely certain, fairly certain, or uncertain.

Chemotherapy recommendation pre-RS was given according to ESMO/ASCO guidelines (depending on country); participants could use (but this was not mandatory) a clinical risk score calculator to help in decision making.

### Study objectives

The primary objective of the study was to assess both oncologists’ degree of confidence and intra- and inter-oncologist agreement in adjuvant chemotherapy treatment recommendation pre- and post-RS.

### Statistics

Descriptive statistics were used to summarize patient and tumor characteristics. Each patient was assigned an identification (ID) number. McNemar’s test was used to assess whether the proportion of patients for whom chemotherapy was recommended changed from pre- to post‐RS. Intra- and inter-observer agreement was assessed by Cohen’s kappa (K) in all possible pairwise comparisons between two oncologists, the Fleiss’ kappa (FK) agreement was used to measure overall agreement in recommendations pre- and post-RS.

### Ethics

The study was conducted in accordance with the Declaration of Helsinki as revised in 2013. All participants signed informed consent to allow use of routine surgical pathology specimens for Oncotype DX testing. The 30 patients included in this study were randomly selected among the cohort of patients participating at the observational PONDx study^31^. The protocol was approved by the Institutional Review Board of San Raffaele Hospital.

### Reporting summary

Further information on research design is available in the Nature Research Reporting Summary linked to this article.

## Supplementary information

Supplementary files

Reporting Summary

## Data Availability

The datasets used and/or analyzed during the current study available from the corresponding author on reasonable request.
