# Supplementary material for: Oncotype DX results increase concordance in adjuvant chemotherapy recommendations for early-stage breast cancer
Source: NPJ Breast Cancer. 2023 Jun 8;9:51. doi: 10.1038/s41523-023-00559-6 (PMC10250312; doi:10.1038/s41523-023-00559-6)
Supplement: Supplementary file 1 — Supplementary files [file 41523_2023_559_MOESM1_ESM.pdf]

Supplementary Figure 1. Change of therapy indication after RS

a) Therapeutic recommendations pre- and post- RS per patient. **b)** Rate and type of therapeutic indication change post-RS results by oncologist country and experience.

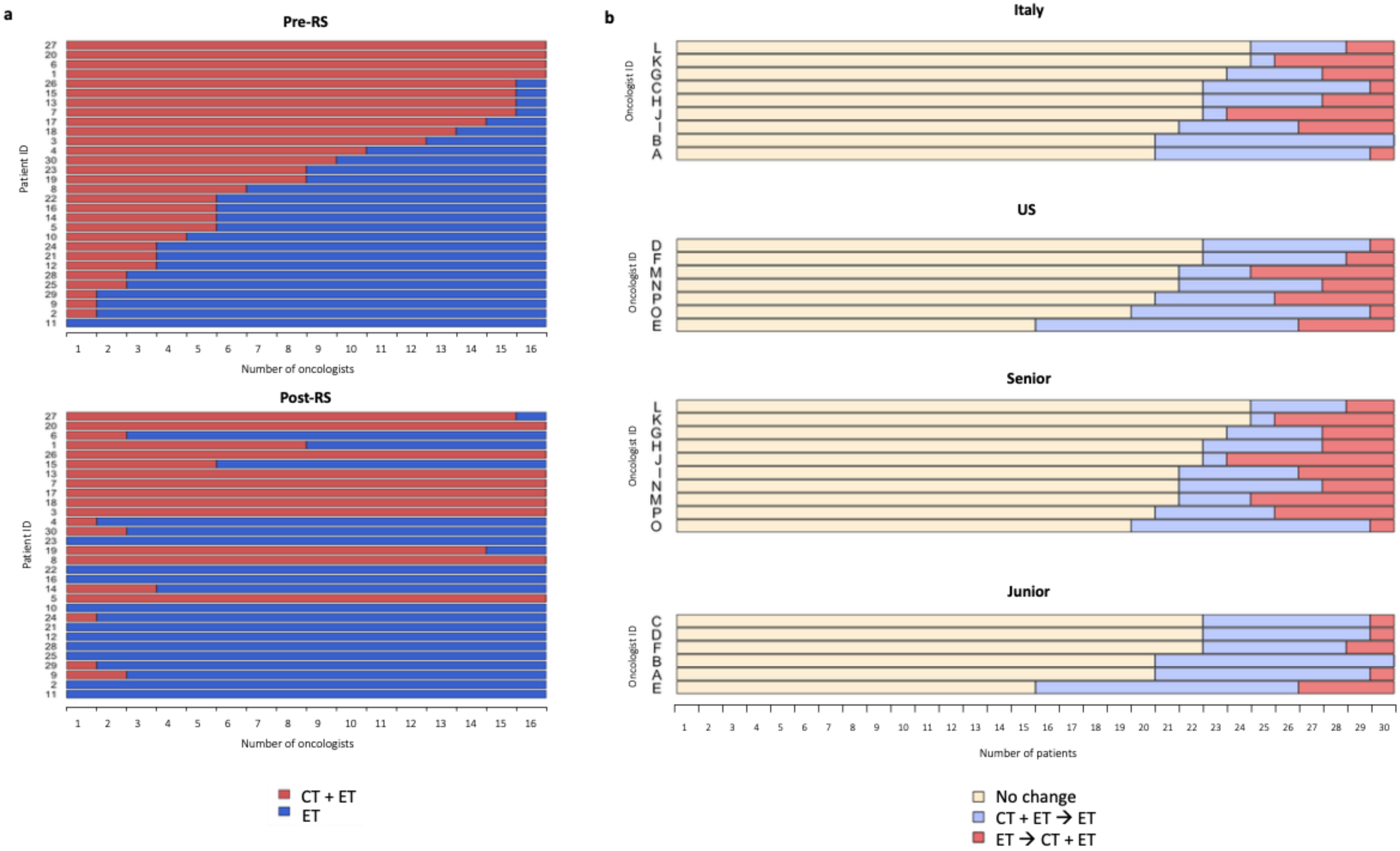

Supplementary Figure 2. Change in the degree of confidence in treatment recommendation pre- and post- RS results by oncologist country and experience

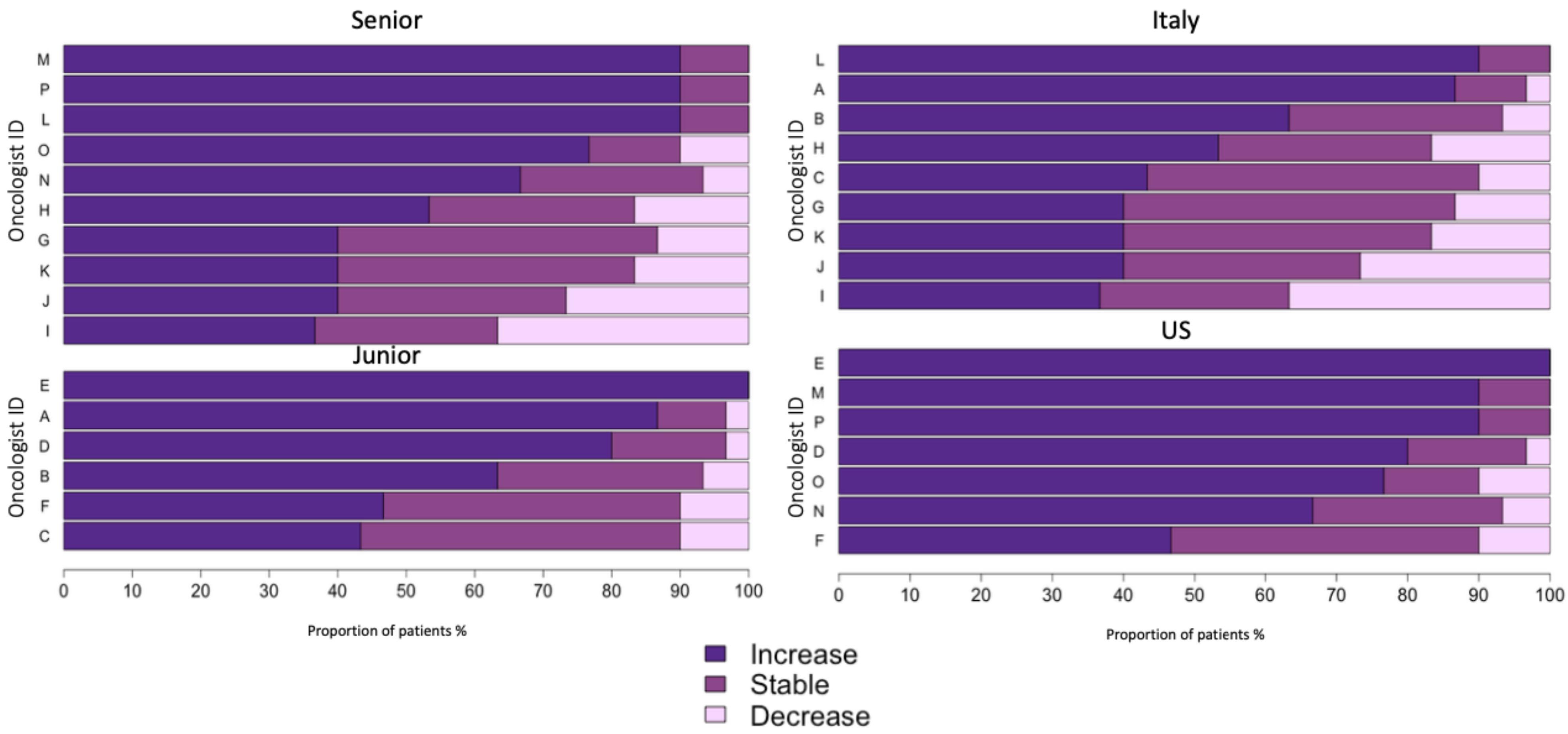

Supplementary Table 1. Clinicopathologic variables and RS results of the 30 patients

ID	Age (range)	Menopausal status	Histotype	Stage	pT	Grade	Ki67	ER	PgR	RS*
1	41-45	Pre/peri-menopausal	Ductal	pT2	2,2	3	34	90	90	15
2	66-70	Post-menopausal	Ductal	pT1c	1,7	2	26	90	80	15
3	56-60	Post-menopausal	Lobular	pT2	2,5	3	37	90	20	41
4	61-65	Post-menopausal	Ductal	pT1c	1,1	2	29	90	0	21
5	51-55	Post-menopausal	Ductal	pT1c	2,0	2	19	90	10	32
6	36-40	Pre/peri-menopausal	Ductal	pT1c	1,6	2	50	95	95	2
7	56-60	Post-menopausal	Ductal	pT1c	1,2	3	31	90	1	39
8	41-45	Pre/peri-menopausal	Ductal	pT1c	1,9	2	28	90	50	52
9	66-70	Post-menopausal	Ductal	pT1c	1,5	2	27	90	90	23
10	46-50	Post-menopausal	Lobular	pT1c	1,5	3	28	90	90	9
11	71-75	Post-menopausal	Mucinous	pT2	2,8	2	16	80	5	24
12	66-70	Post-menopausal	Lobular	pT2	2,3	2	24	90	5	23
13	46-50	Pre/peri-menopausal	Ductal	pT1c	1,7	2	29	90	10	29
14	66-70	Post-menopausal	Ductal	pT2	2,8	2	28	90	90	25
15	46-50	Post-menopausal	Ductal	pT1c	1,4	3	49	90	70	15
16	61-65	Post-menopausal	Lobular	pT2	3,2	2	26	90	70	13
17	51-55	Post-menopausal	Ductal	pT2	2,5	2	32	90	20	29
18	36-40	Pre/peri-menopausal	Ductal	pT1c	1,8	3	28	95	90	37
19	41-45	Pre/peri-menopausal	Ductal	pT2	2,1	2	26	90	80	24
20	26-30	Pre/peri-menopausal	Ductal	pT2	3,0	2	29	90	1	41
21	46-50	Pre/peri-menopausal	Ductal	pT2	2,1	2	22	90	90	10
22	36-40	Pre/peri-menopausal	Ductal	pT1c	1,1	2	25	90	80	9
23	41-45	Pre/peri-menopausal	Ductal	pT1c	1,6	2	30	90	90	11
24	71-75	Post-menopausal	Ductal	pT2	2,5	2	27	90	20	19
25	46-50	Pre/peri-menopausal	Ductal	pT2	2,2	2	21	90	90	9
26	46-50	Post-menopausal	Ductal	pT2	2,4	3	35	90	40	29

27	41-45	Pre/peri-menopausal	Ductal	pT1c	1,3	3	48	90	90	27
28	66-70	Post-menopausal	Ductal	pT2	2,6	2	19	90	10	22
29	51-55	Post-menopausal	Ductal	pT1c	1,1	2	24	90	30	17
30	56-60	Post-menopausal	Ductal	pT2	3,0	2	23	90	5	21

*This column was added only for the second contact
