# Supplementary material for: Oncotype DX results increase concordance in adjuvant chemotherapy recommendations for early-stage breast cancer
Source: NPJ Breast Cancer. 2023 Jun 8;9:51. doi: 10.1038/s41523-023-00559-6 (PMC10250312; doi:10.1038/s41523-023-00559-6)
Supplement: Supplementary file 2 — Reporting Summary [file 41523_2023_559_MOESM2_ESM.pdf]

nature portfolio

Corresponding author(s): Giampaolo Bianchini

Last updated by author(s): May 19, 2023

Reporting Summary

Nature Portfolio wishes to improve the reproducibility of the work that we publish. This form provides structure for consistency and transparency in reporting. For further information on Nature Portfolio policies, see our Editorial Policies and the Editorial Policy Checklist.

Statistics

For all statistical analyses, confirm that the following items are present in the figure legend, table legend, main text, or Methods section.

n/a	Confirmed	
☐	☒	The exact sample size (*n*) for each experimental group/condition, given as a discrete number and unit of measurement
☒	☐	A statement on whether measurements were taken from distinct samples or whether the same sample was measured repeatedly
☐	☒	The statistical test(s) used AND whether they are one- or two-sided<br>*Only common tests should be described solely by name; describe more complex techniques in the Methods section.*
☒	☐	A description of all covariates tested
☒	☐	A description of any assumptions or corrections, such as tests of normality and adjustment for multiple comparisons
☐	☒	A full description of the statistical parameters including central tendency (e.g. means) or other basic estimates (e.g. regression coefficient) AND variation (e.g. standard deviation) or associated estimates of uncertainty (e.g. confidence intervals)
☐	☒	For null hypothesis testing, the test statistic (e.g. F, t, r) with confidence intervals, effect sizes, degrees of freedom and P value noted<br>*Give P values as exact values whenever suitable.*
☒	☐	For Bayesian analysis, information on the choice of priors and Markov chain Monte Carlo settings
☒	☐	For hierarchical and complex designs, identification of the appropriate level for tests and full reporting of outcomes
☒	☐	Estimates of effect sizes (e.g. Cohen's *d*, Pearson's *r*), indicating how they were calculated

Our web collection on statistics for biologists contains articles on many of the points above.

Software and code

Policy information about availability of computer code

Data collection	Excel version 16.0
Data analysis	R version 4.0.2

For manuscripts utilizing custom algorithms or software that are central to the research but not yet described in published literature, software must be made available to editors and reviewers. We strongly encourage code deposition in a community repository (e.g. GitHub). See the Nature Portfolio guidelines for submitting code & software for further information.

Data

Policy information about availability of data

All manuscripts must include a data availability statement. This statement should provide the following information, where applicable:

- Accession codes, unique identifiers, or web links for publicly available datasets
- A description of any restrictions on data availability
- For clinical datasets or third party data, please ensure that the statement adheres to our policy

All data generated or analysed during this study are included in this published article and its supplementary information files.

Human research participants

Policy information about studies involving human research participants and Sex and Gender in Research.

Reporting on sex and gender	Female breast cancer patients
Population characteristics	Patients with ER+/HER2- early breast cancer who underwent breast surgery at San Raffaele Hospital in Milan, Italy
Recruitment	Retrospective analysis of real-world data retrieved from the institutional database of San Raffaele Hospital in Milan, Italy
Ethics oversight	All participants signed informed consent to allow use of routine surgical pathology specimens for Oncotype DX testing

Note that full information on the approval of the study protocol must also be provided in the manuscript.

Field-specific reporting

Please select the one below that is the best fit for your research. If you are not sure, read the appropriate sections before making your selection.

☒ Life sciences ☐ Behavioural & social sciences ☐ Ecological, evolutionary & environmental sciences

For a reference copy of the document with all sections, see nature.com/documents/nr-reporting-summary-flat.pdf

Life sciences study design

All studies must disclose on these points even when the disclosure is negative.

Sample size	No sample-size calculation was performed. We randomly selected 30 clinical cases for this survey study
Data exclusions	Among patients with ER+/HER2- early breast cancer who underwent breast surgery at our institution, we identified those with stage pT1c-2, node-negative, grade 2/3, Ki67 of at least 15%, and RS available
Replication	This study does not contain experimental findings
Randomization	n/a
Blinding	n/a

Reporting for specific materials, systems and methods

We require information from authors about some types of materials, experimental systems and methods used in many studies. Here, indicate whether each material, system or method listed is relevant to your study. If you are not sure if a list item applies to your research, read the appropriate section before selecting a response.

Materials & experimental systems

n/a	Involved in the study	
☒	☐	Antibodies
☒	☐	Eukaryotic cell lines
☒	☐	Palaeontology and archaeology
☒	☐	Animals and other organisms
☐	☒	Clinical data
☒	☐	Dual use research of concern

Methods

n/a	Involved in the study	
☒	☐	ChIP-seq
☒	☐	Flow cytometry
☒	☐	MRI-based neuroimaging

Clinical data

Policy information about clinical studies

All manuscripts should comply with the ICMJE guidelines for publication of clinical research and a completed CONSORT checklist must be included with all submissions.

Clinical trial registration	n/a
Study protocol	n/a

Data collection	The study used real-world data retrieved from the institutional database of San Raffaele Hospital in Milan, Italy. The study was conducted between January and March 2020.
Outcomes	The primary objective of the study was to assess both oncologists' degree of confidence and intra- and inter-oncologists' agreement in adjuvant chemotherapy treatment recommendation pre- and post-RS . McNemar's test was used to assess whether the proportion of patients recommended chemotherapy changed from pre- to post-RS. Intra- and inter-observer agreement was assessed by Cohen's kappa (K) in all possible pairwise comparisons between two oncologists, the Fleiss' kappa (FK) agreement was used to measure overall agreement in recommendations pre- and post-RS
